# Draft Genome Sequence of Plant Growth-Promoting Bacillus altitudinis Strain PAE4

**DOI:** 10.1128/MRA.00962-18

**Published:** 2018-10-04

**Authors:** Nahed Abdel Ghaffar Abdel Aziz Ibrahim, Mohamed Nabil Abdel Mageed Omar, Ghada A. Abu El Heba, Yvan Moënne-Loccoz, Claire Prigent-Combaret, Daniel Muller

**Affiliations:** a Agricultural Genetic Engineering Research institute, ARC, Giza, Egypt; b Soil, Water and Environment Institute, ARC, Giza, Egypt; c CNRS, Université de Lyon, Université Claude Bernard Lyon 1, INRA, VetAgro Sup, UMR 5557 Ecologie Microbienne, Villeurbanne, France; University of Maryland School of Medicine

## Abstract

We report here the draft genome of Bacillus altitudinis strain PAE4, a thermophilic plant growth-promoting rhizobacterium isolated from the coastal ridge of the Mediterranean Sea in Egypt. Besides heat shock protein genes, several genes encoding phytobeneficial properties were identified.

## ANNOUNCEMENT

Bacillus altitudinis is a species encompassing strains that interact often with plants. Most studied strains are well described, as plant growth-promoting rhizobacteria (PGPRs) are known for their phytostimulation abilities (by producing phytohormones or enhancing mineral nutrition [1]), their biocontrol activities (by eliciting plant immune systemic responses or directly inhibiting phytopathogens [2]), and even their ability to help plants in phytoremediation systems (3).

B. altitudinis PAE4 is a bacterial strain isolated from the coastal Mediterranean Ridge in Egypt (4). Strain PAE4 is thermophilic, as it can grow in temperatures up to 70°C (4). As an inoculum, it improved maize and wheat grain yield in field conditions (5). The PII protein (gene *glnK*) that regulates nitrogen metabolism and the phosphotransferase system (PTS) interconnecting carbon and nitrogen metabolisms was shown to play a crucial role in its phytobeneficial activity (5).

Genomic DNA extraction was done from an overnight culture in LB medium (6) at 37°C using a Nucleospin tissue kit (catalog number 740952.50; Macherey-Nagel, Hoerdt, France). Genomic DNA was sequenced at MR DNA (Shallowater, TX, USA) using Illumina MiSeq technology, generating a 2 × 300-bp paired-end library. NGen version 14 software (DNAStar, Inc.) was used for trimming sequences (default settings) and for *de novo* assembly (the average size of B. altitudinis genomes was used as an assembly parameter). Genome annotation was done with the MicroScope platform version 3.10.0 (7, 8).

A total of 5,940,024 paired-end reads were obtained, giving a coverage depth of 482×. The resulting assemblies generated 28 contigs. The maximum length and *N*_50_ values of the contigs were 962,863 bp and 877,514 bp, respectively, giving a genome 3,693,314 bp in size with a 36.39% G+C content.

The draft genome harbors 3 rRNAs (1 16S rRNA, 1 23S rRNA, and 1 5S rRNA gene), 70 tRNAs, 2,458 protein-coding genes with functional predictions, and 1,469 genes coding for hypothetical proteins. The *rrs* sequence identity was over 99% with B. pumilus and B. altitudinis. The Genome-to-Genome Distance Calculator version 2.1 (GGDC2.1) (9) gave a DNA-DNA hybridization estimate of 90.8% with B. altitudinis strain 41KF2b^T^, showing that PAE4 belongs to the B. altitudinis species.

Besides general metabolism genes that were shown to be involved in the phytostimulation behavior of PAE4 (such as *glnK* and its downstream regulated genes coding for glutamine synthase, ammonium transport, and phosphoenolpyruvate carboxylase), several genes were present for the PAE4 genome code functions that are more specifically involved in plant-microbe interactions (10, 11), such as auxin and cellulose production and xylanase, pectinesterase, amidase, and quercitin dioxygenase activities (12, 13), and that may be important for phytostimulation. In addition, PAE4 possesses a complete type VII secretion system for cell-to-cell molecular communication (14). Six biosynthesis gene clusters for secondary metabolites were predicted with antiSMASH version 4.0.2 (15) in the PAE4 genome. Two clusters are involved in spore coat component biosynthesis. Three other clusters are implicated in the biosynthesis of compounds with anti-phytopathogen activity (fungal or bacterial), i.e., bacilysin, surfactin, and alkylresorsinol (16, 17). Further experiments of plant protection and growth promotion will be performed to clarify the plant-beneficial potential of this bacterium.

### Data availability.

This draft genome sequence has been deposited at GenBank/ENA under BioProject number PRJEB26379, BioSample number ERS2486119, and SRA accession number ERX2730420. Contig sequences of the *de novo* assembly have been deposited at EMBL under the accession numbers OVSL01000001 to OVSL01000028.
